# Supplementary material for: An efficient CRISPR-based strategy to insert small and large fragments of DNA using short homology arms
Source: eLife. 2019 Nov 1;8:e51539. doi: 10.7554/eLife.51539 (PMC6855806; doi:10.7554/eLife.51539)
Supplement: Supplementary file 1. [file elife-51539-supp1.docx]

**Sequence of the attP-SA-Linker-sfGFP-Linker-SD-attP (phase 0)**

GAATTCTGTAAAACGACGGCCAGTGGAGTAGTGCCCCAACTGGGGTAACCTTTGAGTTCTCTCAGTTGGGGGCGTAGGCCTGCAGGAGTCGATCCAACATGGCGACTTGTCCCATCCCCGGCATGTTTAAATATACTAATTATTCTTGAACTAATTTTAATCAACCGATTTATCTCTCTTCCGCAGGTGGGAGGTTCCGGTGGAAGCGGAGGTAGCGGCGGATCCATGGTGTCCAAGGGCGAGGAGCTGTTCACCGGCGTGGTGCCCATCCTGGTGGAGCTGGATGGCGACGTGAACGGCCACAAGTTCAGCGTGCGCGGCGAGGGCGAGGGCGACGCCACCAACGGCAAGCTGACCCTGAAGTTCATCTGCACCACCGGCAAGCTGCCCGTGCCCTGGCCCACCCTGGTGACCACCCTGACCTACGGCGTGCAGTGCTTCAGCCGCTACCCCGATCACATGAAGCAGCACGATTTCTTCAAGAGCGCCATGCCCGAGGGCTACGTGCAGGAGCGCACCATCAGCTTCAAGGATGACGGCACCTACAAGACCCGCGCCGAGGTGAAGTTCGAGGGCGATACCCTGGTGAACCGCATCGAGCTGAAGGGCATCGATTTCAAGGAGGATGGCAACATCCTGGGCCACAAGCTGGAGTACAACTTCAACAGCCACAACGTGTACATCACCGCCGATAAGCAGAAGAACGGCATCAAGGCCAACTTCAAGATCCGCCACAATGTGGAGGATGGCTCCGTGCAGCTGGCCGATCACTACCAGCAGAACACCCCCATCGGCGACGGCCCAGTGCTGCTGCCCGATAACCACTACCTGAGCACCCAGAGCGTGCTGTCCAAGGACCCCAACGAGAAGCGCGATCACATGGTGCTGCTGGAGTTCGTGACCGCCGCCGGCATCACCCTGGGCATGGATGAGCTGTACAAGGGTACCGGATCCGGAGGTAGCGGTGGAAGCGGAGGTTCCGGCGAGGTAAGTTATTGAACAATGGCATCAAATGCCTTCATCATCACTACCCTTTAGCCCTTAAGACCCCACAATGACCTTACCCACTCAGAGAAAAAAGTAAATATGAAAGCCCATTTGAACTTCTGCGGCCGCcctacgcccccaactgagagaactcaaaggttaccccagttggggcactactccgtcatagctgtttcctgggggc

NNNN primer binding site; NNNN phiC31 attP; NNNN *mhc* splice acceptor; NNNN Linker sequence; NNNN sfGFP; NNNN *mhc* splice donor; nnnn reverse strand

Linker sequence for phase 1: GTGGCGGAGGTTCCGGTGGAAGCGGAGGTAGCGGC //

GGTACCGGATCCGGAGGTAGCGGTGGAAGCGGAGGTTCCGGAG

Linker sequence for phase 2: GTCGGGAGGTTCCGGTGGAAGCGGAGGTAGCGGC //

GGTACCGGATCCGGAGGTAGCGGTGGAAGCGGAGGTTCCGGCAG

**Sequence of the attP-3XP3-EGFP-attP**

GAATTCTGTAAAACGACGGCCAGTGGAGTAGTGCCCCAACTGGGGTAACCTTTGAGTTCTCTCAGTTGGGGGCGTAGGGGATCTAATTCAATTAGAGACTAATTCAATTAGAGCTAATTCAATTAGGATCCAAGCTTATCGATTTCGAACCCTCGACCGCCGGAGTATAAATAGAGGCGCTTCGTCTACGGAGCGACAATTCAATTCAAACAAGCAAAGTGAACACGTCGCTAAGCGAAAGCTAAGCAAATAAACAAGCGCAGCTGAACAAGCTAAACAATCGGACTAGAGCCGGTCGCCGGCCGGCCACCATGGTGTCCAAGGGCGAGGAGCTGTTCACCGGCGTGGTGCCAATTCTGGTGGAGCTGGATGGCGACGTGAACGGCCACAAGTTCAGCGTGTCCGGCGAGGGCGAGGGCGACGCCACCTATGGAAAGCTGACCCTGAAGTTCATCTGCACCACCGGCAAGCTGCCCGTGCCATGGCCAACCCTCGTGACCACCCTGACCTATGGCGTGCAGTGCTTCAGCCGCTACCCCGATCACATGAAGCAGCACGATTTCTTCAAGAGCGCCATGCCCGAGGGCTACGTGCAGGAGCGCACCATCTTTTTCAAGGATGACGGCAACTACAAGACCCGCGCCGAAGTGAAGTTCGAGGGCGATACCCTCGTGAACCGCATCGAGCTGAAGGGCATCGATTTCAAGGAGGATGGAAACATCCTGGGCCACAAGCTGGAGTACAACTACAACAGCCACAACGTGTACATCATGGCCGACAAGCAGAAGAACGGCATCAAGGCCAACTTCAAGATCCGCCACAACATCGAGGATGGCGGCGTGCAGCTGGCCGATCACTACCAGCAGAACACCCCAATCGGCGACGGCCCAGTGCTGCTGCCCGATAACCATTACCTGAGCACCCAGAGCGCCCTGAGCAAGGATCCCAACGAGAAGCGCGACCACATGGTGCTGCTGGAGTTTGTGACCGCCGCCGGCATTACCCTGGGCATGGATGAGCTGTACAAGTAGGATCCAGACATGATAAGATACATTGATGAGTTTGGACAAACCACAACTAGAATGCAGTGAAAAAAATGCTTTATTTGTGAAATTTGTGATGCTATTGCTTTATTTGTAACCATTATAAGCTGCAATAAACAAGTTcctacgcccccaactgagagaactcaaaggttaccccagttggggcactactccgtcatagctgtttcctggcggc

NNNN primer binding site; NNNN phiC31 attP; NNNN 3XP3GFP-polyA;

nnnn reverse strand

**Sequence of the gRNA1 target-Homology arm-attP-SA-3XSTOP-minipolyA-U6gRNA1-attP-Homology arm-gRNA1 target**

GTAGTACGATCATAACAACGCGGNNNNNNNNNNNNNNNNNNNNNNNNNNNNNNNNNNNNNNNNNNNNNNNNNNNNNNNNNNNNNNNNNNNNNNNNNNNNNNNNNNNNNNNNNNNNNNNNNNNNGGAGTAGTGCCCCAACTGGGGTAACCTTTGAGTTCTCTCAGTTGGGGGCGTAGGCCTGCAGGAGTCGATCCAACATGGCGACTTGTCCCATCCCCGGCATGTTTAAATATACTAATTATTCTTGAACTAATTTTAATCAACCGATTTATCTCTCTTCCGCAGGTTAACGTAACCTAGGAAATAAAATACGAAATGAATTCTACCCAAAAGCAGAGAGGGCGCCAGTGCTCACTACTTTTTATAATTCTCAACTTCTTTTTCCAGACTCAGTTCGTATATATAGACCTATTTTCAATTTAACGTCGTAGTACGATCATAACAACGGTTTTAGAGCTAGAAATAGCAAGTTAAAATAAGGCTAGTCCGTTATCAACTTGAAAAAGTGGCACCGAGTCGGTGCTTTTTTTGCGGCCGCcctacgcccccaactgagagaactcaaaggttaccccagttggggcactactccNNNNNNNNNNNNNNNNNNNNNNNNNNNNNNNNNNNNNNNNNNNNNNNNNNNNNNNNNNNNNNNNNNNNNNNNNNNNNNNNNNNNNNNNNNNNNNNNNNNNccgcgttgttatgatcgtactac

NNNN gRNA1-target; NNNN Left homology arm; NNNN phiC31 attP; NNNN *mhc* splice acceptor; NNNN Stop codons; NNNN short polyA; NNNN U6 promoter-gRNA1 Dominant marker ; NNNN Right homology arm; nnnn reverse strand

**Sequence of the gRNA1 target-Homology arm-attP-SA-T2A-miniGAL4-miniPA-U6gRNA1-attP-Homology arm-gRNA1 target (phase 0)**

GTAGTACGATCATAACAACGCGGNNNNNNNNNNNNNNNNNNNNNNNNNNNNNNNNNNNNNNNNNNNNNNNNNNNNNNNNNNNNNNNNNNNNNNNNNNNNNNNNNNNNNNNNNNNNNNNNNNNGAATTCTGTAAAACGACGGCCAGTGGAGTAGTGCCCCAACTGGGGTAACCTTTGAGTTCTCTCAGTTGGGGGCGTAGGCCTGCAGGAGTCGATCCAACATGGCGACTTGTCCCATCCCCGGCATGTTTAAATATACTAATTATTCTTGAACTAATTTTAATCAACCGATTTATCTCTCTTCCGCAGGTGGGAGGTTCCGGTGGAAGCGGAGGTAGCGGCGGATCCGAGGGCCGCGGCAGCCTGCTGACCTGCGGCGATGTGGAGGAGAACCCCGGGCCCATGAAGCTGTTGTCCTCCATCGAGCAAGCTTGTGACATCTGCCGTTTAAAGAAGCTGAAGTGCAGCAAGGAGAAGCCCAAATGCGCTAAGTGTTTAAAGAACAATTGGGAATGCCGCTACAGCCCCAAGACCAAGCGCAGCCCCTTGACCCGCGCTCATTTAACCGAAGTCGAGAGCCGTTTAGAGCGCTTGGAGCAACTGTTTTTACTGATCTTTCCCCGCGAGGATTTAGACATGATTTTAAAGATGGACTCTTTACAAGATATCAAGGCTTTACTGACCGGCTTGTTCGTGCAAGATAACGTGAATAAGGATGCCGTGACCGACCGTTTAGCTTCCGTGGAAACTGATATGCCTTTAACTTTACGTCAGCATCGTATCTCCGCCACCAGCTCCTCCGAGGAAAGCAGCAACAAGGGCCAGCGCCAGTTGACCGTGTCCATCGATAGCGCCGCCCACCACGACAACTCCACCATTCCGCTGGACTTCATGCCCCGCGATGCTTTACACGGATTCGACTGGTCCGAGGAGGATGATATGTCCGACGGTTTACCCTTTTTAAAAACCGACCCCAACAACAACGGCTTCTTTGGCGATGGCTCTTTATTGTGCATTTTACGCTCCATCGTCAAGCTGCTGTCCAATCGCCCCCCGTCCCGCAACAGCCCCGTGACCATTCCGCGCAGCACCCCCAGCCATCGCTCCGTCACCCCGTTCTTGGGCCAGCAGCAGCAGCTGCAATCTTTAGTGCCGTTGACGCCCAGCGCTTTATTTGGCGGAGCCAATTTCAATCAGAGCGGCAACATCGCCGACAGCTCTTTATCCTTCACCTTCACCAACTCCAGCAACGGCCCGAATTTAATCACCACGCAGACCAACAGCCAAGCTTTAAGCCAGCCGATTGCCTCCTCCAACGTCCACGACAACTTCATGAACAACGAGATTACCGCCTCCAAGATCGATGACGGCAATAATTCCAAGCCTTTATCCCCGGGATGGACGGACCAAACCGCCTACAATGCCTTCGGCATCACCACGGGCATGTTCAACACCACCACCATGGACGACGTGTACAACTATTTATTCGATGACGAGGATACCCCCCCGAACCCCAAAAAGGAGTAACCTAGGAAATAAAATACGAAATGAATTCTACCCAAAAGCAGAGAGGGCGCCAGTGCTCACTACTTTTTATAATTCTCAACTTCTTTTTCCAGACTCAGTTCGTATATATAGACCTATTTTCAATTTAACGTCGTAGTACGATCATAACAACGGTTTTAGAGCTAGAAATAGCAAGTTAAAATAAGGCTAGTCCGTTATCAACTTGAAAAAGTGGCACCGAGTCGGTGCTTTTTTTGCGGCCGCcctacgcccccaactgagagaactcaaaggttaccccagttggggcactactccgtcatagctgtttcctgggggcNNNNNNNNNNNNNNNNNNNNNNNNNNNNNNNNNNNNNNNNNNNNNNNNNNNNNNNNNNNNNNNNNNNNNNNNNNNNNNNNNNNNNNNNNNNNNNNNNNNccgcgttgttatgatcgtactac

NNNN gRNA1-target; NNNN Left homology arm; NNNN primer binding site; NNNN phiC31 attP; NNNN *mhc* splice acceptor; NNNN Linker sequence; NNNN T2A- miniGAL4; NNNN short polyA; NNNN U6 promoter-gRNA1 Dominant marker NNNN Right homology arm; nnnn reverse strand

Linker sequence for phase 1: TGGGAGGTTCCGGTGGAAGCGGAGGTAGCGGC

Linker sequence for phase 2: GTCGGGAGGTTCCGGTGGAAGCGGAGGTAGCGG C

**Sequence of the Int100-Scaffold**

GTAGTACGATCATAACAACGCGGNNNNNNNNNNNNNNNNNNNNNNNNNNNNNNNNNNNNNNNNNNNNNNNNNNNNNNNNNNNNNNNNNNNNNNNNNNNNNNNNNNNNNNNNNNNNNNNNNNNNAAACTTGTCTTCATTATATAGAAGACTTCGCGNNNNNNNNNNNNNNNNNNNNNNNNNNNNNNNNNNNNNNNNNNNNNNNNNNNNNNNNNNNNNNNNNNNNNNNNNNNNNNNNNNNNNNNNNNNNNNNNNNNNccgcgttgttatgatcgtactac

NNNN gRNA1_-arget; NNNN Left homology arm; NNNN Right homology arm; NNNN RE Cassette ; nnnn reverse strand
