# Supplementary material for: An efficient CRISPR-based strategy to insert small and large fragments of DNA using short homology arms
Source: eLife. 2019 Nov 1;8:e51539. doi: 10.7554/eLife.51539 (PMC6855806; doi:10.7554/eLife.51539)
Supplement: Supplementary file 2. [file elife-51539-supp2.docx]

**Design of Int-100 CRIMIC constructs:**

Design region:

**
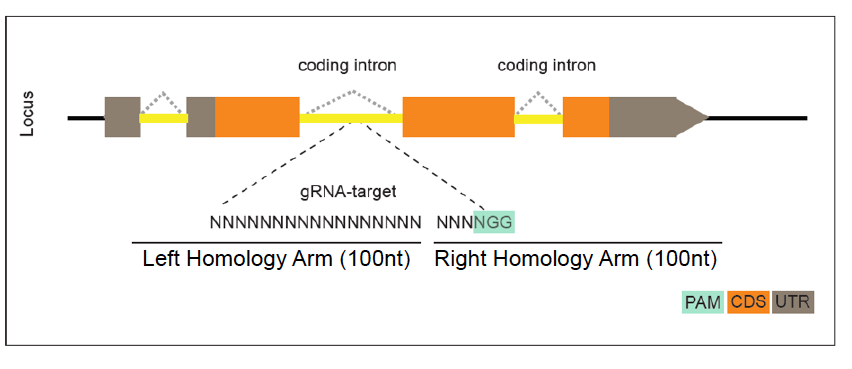
**

- 100 nucleotides on both sides of the cut site are used for synthesis using the template DNA file (int100_scaffold file, see above)

After synthesis the construct looks like below:


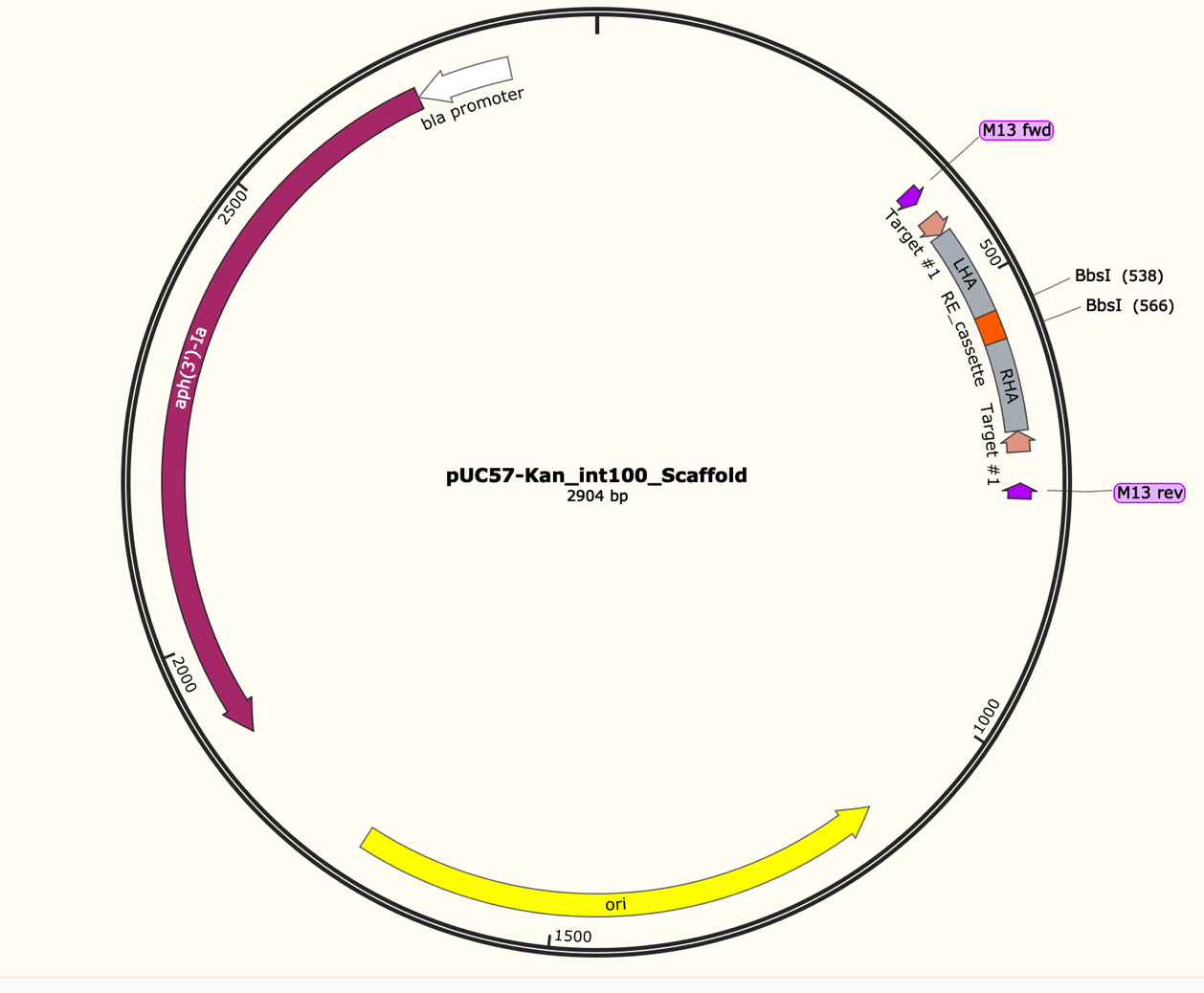


**Cloning of ds drop-in constructs:**

- Cut the vector with BbsI-HF (NEB R3539L). Cut ~2ug of plasmid for 2hrs at 37℃ in 30 ul reaction volume with 1 ul of enzyme (~20Units).

-Cut pM37 of proper phase with BbsI-HF and ScaI-HF (ScaI-HF cuts the pM37 backbone to help resolve the fragments easier (below is how BbsI alone vs BbsI+ScaI looks). If you do not wish to cut with ScaI-HF resolving the gel longer would work too). 4848 bp fragment is the one to isolate.


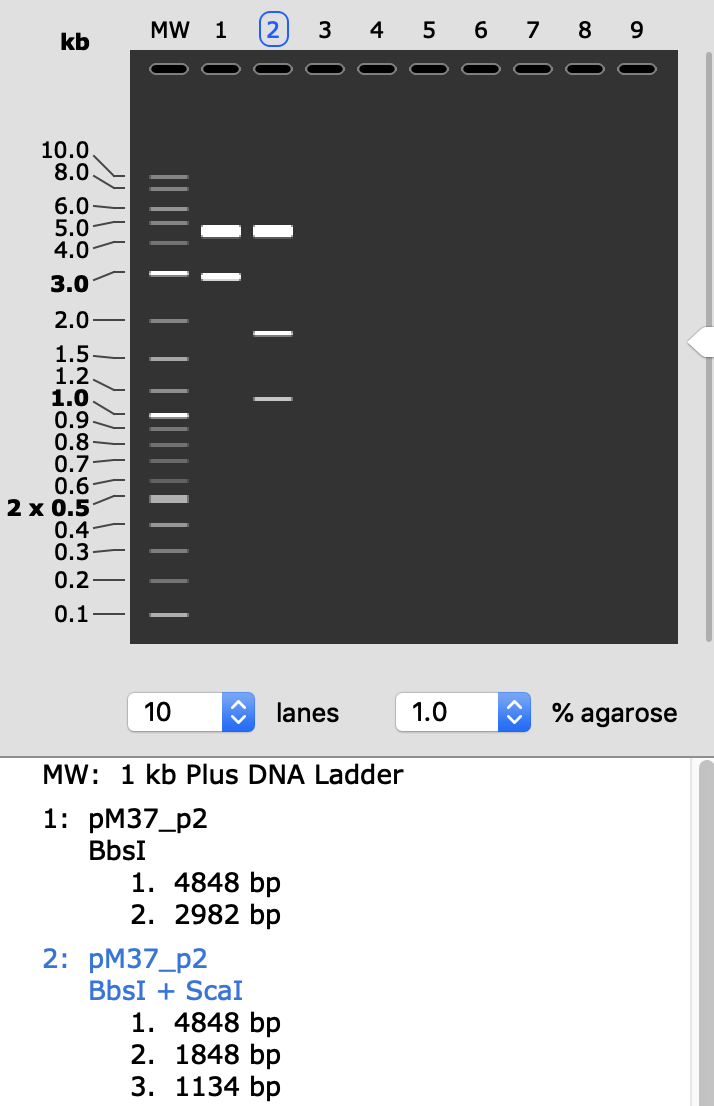


- Gel isolate fragments and set up ligation reaction:

100ng vector backbone

150-200 ng pM37 fragment

1ul T4 ligase buffer

1ul T4 ligase (NEB M0202L, not quick ligase)

dH_2_O to 10ul

Incubate 1-2 hrs at RT. Transform 1ul to 50 ul chemocompetent DH5-alpha. Selection antibiotic is Kanamycin, hence 1-hour recovery is necessary after heatshock. Plate on LB plates with Kanamycin. Incubate at 37℃ overnight.

-Next day do colony PCR with primers M13F-Long for CRIMIC-ch-rev

M13F-long for gacgttgtaaaacgacggccag

CRIMIC-ch-rev gcggaagagagataaatcggttg

Use autoclaved micropipette tip to pick a colony, touch it on a gridded plate to copy the colony and dip the same pipette tip to PCR mix.

PCR conditions

0.2ul forward primer

0.2ul reverse primer

12.1 ul dH_2_O

12.5 ul OneTaq Quick-Load 2X Master Mix (M0486L)

Rxn: 94℃ 30 sec

94℃ 30 sec |

58℃ 30 sec | 34 cycles

68℃ 30 sec |

68℃ 5 minutes

8℃ Hold

-The positive colonies will show ~535 nt amplicon. (Example below)

-Positive colonies can be grown for Mini/Midi prep. Final constructs are sequenced using M13 forward/reverse primers.

**
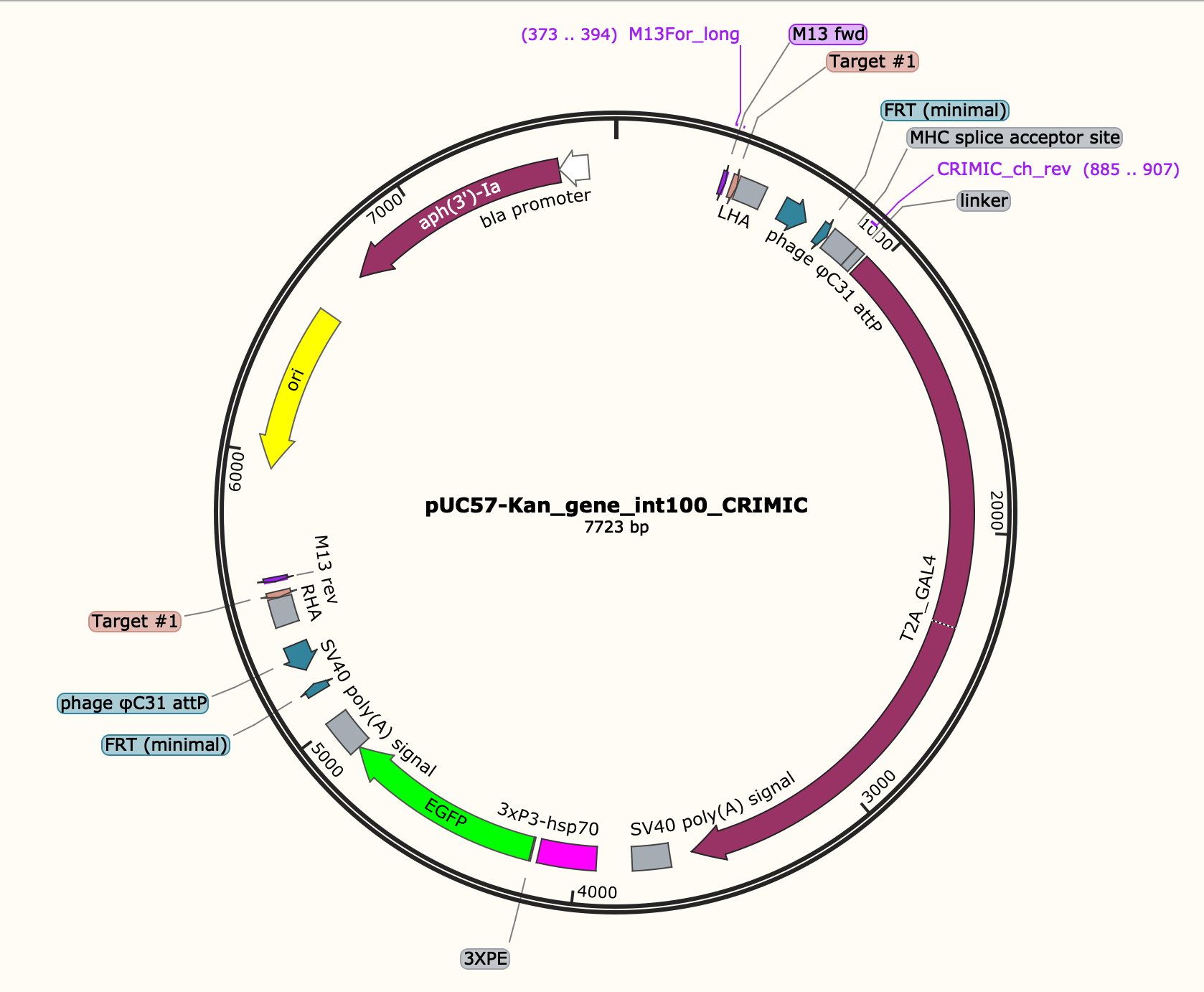
**

**Injection:**

-We inject in ~400 embryos of isogenized nos-Cas9 fly lines (iso6 for X, iso18 for 2^nd^ and iso5 for 3^rd^ chromosome). The WGS for these lines should be used for the design.

-Inject 200-400 ng/ul of donor DNA together with 25ng/ul pCDF3 gene specific gRNA plasmid.
