# Supplementary material for: An efficient CRISPR-based strategy to insert small and large fragments of DNA using short homology arms
Source: eLife. 2019 Nov 1;8:e51539. doi: 10.7554/eLife.51539 (PMC6855806; doi:10.7554/eLife.51539)
Supplement: Transparent reporting form [file elife-51539-transrepform.docx]

***eLife’s* transparent reporting form**

We encourage authors to provide detailed information *within their submission* to facilitate the interpretation and replication of experiments. Authors can upload supporting documentation to indicate the use of appropriate reporting guidelines for health-related research (see [EQUATOR Network](http://www.equator-network.org/%20)), life science research (see the [BioSharing Information Resource](https://biosharing.org/)), or the [ARRIVE guidelines](http://www.plosbiology.org/article/info:doi/10.1371/journal.pbio.1000412) for reporting work involving animal research. Where applicable, authors should refer to any relevant reporting standards documents in this form.

If you have any questions, please consult our Journal Policies and/or contact us: [editorial@elifesciences.org](mailto:editorial@elifesciences.org).

**Sample-size estimation**

- You should state whether an appropriate sample size was computed when the study was being designed
- You should state the statistical method of sample size computation and any required assumptions
- If no explicit power analysis was used, you should describe how you decided what sample (replicate) size (number) to use

Please outline where this information can be found within the submission (e.g., sections or figure legends), or explain why this information doesn’t apply to your submission:

Fly transgenesis experiments are labor intensive by nature. They involve micro-injection of 400-600 embryos individually, selecting the surviving larvae, setting single fly crosses of the resulting flies and establishing independent transgenic lines. On the other hand, targeting only a single locus with a transgenesis method can be misleading due to locus specific effects. Hence, estimation of efficiency of methods that involve transgenesis efficiency typically rely on 5-10 targeted loci, a number empirically found to be meaningful to determine success rate based on our and others experience. Indeed, the success rate of transgenesis we obtained with the CRIMIC method in the 10 selected loci (4/10) was in line with the overall success rate we have obtained while targeting more than 1000 different loci (~50% success rate). The information can be found in the introduction of the manuscript. We typically test 5-10 genes and when the data are quite consistent between experiments ( see data in this manuscript or in past publications (e.g. Li-Kroeger et al., 2018) there are no worries that the method is not generally applicable.

**Replicates**

- You should report how often each experiment was performed
- You should include a definition of biological versus technical replication
- The data obtained should be provided and sufficient information should be provided to indicate the number of independent biological and/or technical replicates
- If you encountered any outliers, you should describe how these were handled
- Criteria for exclusion/inclusion of data should be clearly stated
- High-throughput sequence data should be uploaded before submission, with a private link for reviewers provided (these are available from both GEO and ArrayExpress)

Please outline where this information can be found within the submission (e.g., sections or figure legends), or explain why this information doesn’t apply to your submission:

The injections were conducted once per construct. This is the meaningful success rate for a large-scale gene tagging effort. We reported all the raw data for success rates without eliminating any positive or negative outliers. All the raw data is available in the figures (3,4,6) and table 1.

**Statistical reporting**

- Statistical analysis methods should be described and justified
- Raw data should be presented in figures whenever informative to do so (typically when N per group is less than 10)
- For each experiment, you should identify the statistical tests used, exact values of N, definitions of center, methods of multiple test correction, and dispersion and precision measures (e.g., mean, median, SD, SEM, confidence intervals; and, for the major substantive results, a measure of effect size (e.g., Pearson's r, Cohen's d)
- Report exact p-values wherever possible alongside the summary statistics and 95% confidence intervals. These should be reported for all key questions and not only when the p-value is less than 0.05.

Please outline where this information can be found within the submission (e.g., sections or figure legends), or explain why this information doesn’t apply to your submission:

Statistics and experimental design can be found in materials and methods.

(For large datasets, or papers with a very large number of statistical tests, you may upload a single table file with tests, Ns, etc., with reference to sections in the manuscript.)

**Group allocation**

- Indicate how samples were allocated into experimental groups (in the case of clinical studies, please specify allocation to treatment method); if randomization was used, please also state if restricted randomization was applied
- Indicate if masking was used during group allocation, data collection and/or data analysis

Please outline where this information can be found within the submission (e.g., sections or figure legends), or explain why this information doesn’t apply to your submission:

This does not apply for this study.

**Additional data files (“source data”)**

- We encourage you to upload relevant additional data files, such as numerical data that are represented as a graph in a figure, or as a summary table
- Where provided, these should be in the most useful format, and they can be uploaded as “Source data” files linked to a main figure or table
- Include model definition files including the full list of parameters used
- Include code used for data analysis (e.g., R, MatLab)
- Avoid stating that data files are “available upon request”

Please indicate the figures or tables for which source data files have been provided:

Source data can be found in the figures 3, 4, 6 and table 1.
